# Changes in the Molecular Epidemiology of Pediatric Bacterial Meningitis in Senegal After Pneumococcal Conjugate Vaccine Introduction

**DOI:** 10.1093/cid/ciz517

**Published:** 2019-09-05

**Authors:** Mouhamadou A Sonko, Felix S Dube, Catherine Bi Okoi, Amadou Diop, Aliou Thiongane, Madikay Senghore, Peter Ndow, Archibal Worwui, Papa M Faye, Baidy Dieye, Idrissa D Ba, Aliou Diallo, Diop Boly, Ousmane Ndiaye, Moussa F Cissé, Jason M Mwenda, Brenda A Kwambana-Adams, Martin Antonio

**Affiliations:** 1 Centre Hospitalier National d’Enfants Albert Royer, Dakar, Senegal; 2 World Health Organization Regional Reference Laboratory, Medical Research Council Unit The Gambia at the London School of Hygiene and Tropical Medicine, Fajara; 3 Department of Molecular and Cell Biology, University of Cape Town, South Africa; 4 World Health Organization Country Office, Dakar, Senegal; 5 Ministry of Health, Dakar, Senegal; 6 World Health Organization, Regional Office for Africa, Immunization, Vaccines, and Development, Brazzaville, Congo; 7 Faculty of Infectious and Tropical Diseases, London School of Hygiene and Tropical Medicine, United Kingdom; 8 Microbiology and Infection Unit, Warwick Medical School, University of Warwick, Coventry, United Kingdom

**Keywords:** pneumococcal conjugate vaccines, pediatric bacterial meningitis, *Streptococcus pneumoniae*, *Haemophilus influenzae type* b, *Neisseria meningitidis*

## Abstract

**Background:**

Bacterial meningitis is a major cause of mortality among children under 5 years of age. Senegal is part of World Health Organization–coordinated sentinel site surveillance for pediatric bacterial meningitis surveillance. We conducted this analysis to describe the epidemiology and etiology of bacterial meningitis among children less than 5 years in Senegal from 2010 and to 2016.

**Methods:**

Children who met the inclusion criteria for suspected meningitis at the Centre Hospitalier National d’Enfants Albert Royer, Senegal, from 2010 to 2016 were included. Cerebrospinal fluid specimens were collected from suspected cases examined by routine bacteriology and molecular assays. Serotyping, antimicrobial susceptibility testing, and whole-genome sequencing were performed.

**Results:**

A total of 1013 children were admitted with suspected meningitis during the surveillance period. *Streptococcus pneumoniae*, *Neisseria meningitidis*, and *Haemophilus* accounted for 66% (76/115), 25% (29/115), and 9% (10/115) of all confirmed cases, respectively. Most of the suspected cases (63%; 639/1013) and laboratory-confirmed (57%; 66/115) cases occurred during the first year of life. Pneumococcal meningitis case fatality rate was 6-fold higher than that of meningococcal meningitis (28% vs 5%). The predominant pneumococcal lineage causing meningitis was sequence type 618 (n = 7), commonly found among serotype 1 isolates. An ST 2174 lineage that included serotypes 19A and 23F was resistant to trimethoprim-sulfamethoxazole.

**Conclusions:**

There has been a decline in pneumococcal meningitis post–pneumococcal conjugate vaccine introduction in Senegal. However, disease caused by pathogens covered by vaccines in widespread use still persists. There is need for continued effective monitoring of vaccine-preventable meningitis.

## Teaser

Disease caused by pathogens covered by conjugate vaccines in widespread use still persists in Senegal. There is need for continued effective monitoring of vaccine-preventable meningitis.

Childhood bacterial meningitis remains a significant cause of morbidity and mortality worldwide [1–3]. In 2015 alone, meningitis and sepsis ranked among the top 5 leading causes of mortality, causing an estimated 517 000 under-5 deaths [3, 4]. Sub-Saharan Africa has the largest burden of disease, bearing approximately half of the annual cases of meningitis that occur globally [2, 5, 6]. Beyond the neonatal period, the predominant causes of bacterial meningitis are *Streptococcus pneumoniae* (pneumococcus), *Neisseria meningitidis* (meningococcus), and *Haemophilus influenzae* type b (Hib) [6–11]. The third United Nations Sustainable Development Goals is to ensure healthy lives and promote well-being for all at all ages and targets to reduce under-5 mortality to fewer than 25 per 1000 live births by 2030 [3]. Control of pneumococcus, meningococcus, and Hib is of paramount importance in achieving this goal in sub-Saharan Africa.

Immunization against pneumococcus, meningococcus, and Hib is one of the proven ways of reducing morbidity and mortality of childhood bacterial meningitis [1, 12, 13]. In light of this, initiatives such as the Gavi, the Vaccine Alliance, and the Meningitis Vaccine Project (MVP) have led to the accelerated introduction of pneumococcal, Hib, and meningococcal conjugate vaccines in several African countries, including Senegal, during the last 10–15 years [12]. In Senegal, Hib vaccine was introduced into the routine immunization program for children in 2005 as a primary 3-dose course at 2, 3, and 4 months with no booster. Similarly, the 13-valent pneumococcal conjugate vaccine (PCV-13) was introduced in 2014 without a catch-up in a 3 + 0 schedule, with doses given at 2, 3, and 4 months. Vaccine coverage rates remained high (>90%) for the Hib conjugate vaccine and above 80% for PCV-13 from 2014 to 2016. MenAfricVac mass campaigns targeting meningococcus serogroup A (NmA) were conducted in 2013 for individuals aged 1–29 years. However, assessing the impact of new vaccine is reliant on robust and high-quality surveillance data, which are lacking in many low-income countries including Senegal.

The World Health Organization (WHO) recommends implementation of molecular tools in routine surveillance of invasive bacterial vaccine-preventable diseases globally. In Senegal, this was facilitated by the WHO Regional Reference Laboratory (RRL), Medical Research Council (MRC) Unit The Gambia at the London School of Hygiene and Tropical Medicine. We conducted this analysis to describe the epidemiology and etiology of bacterial meningitis among children less than 5 years of age in Senegal from 2010 and to 2016.

## METHODS

Hospital-based sentinel surveillance of bacterial meningitis is ongoing at the Centre Hospitalier National d’Enfants Albert Royer (CHNEAR) located in Dakar, the capital city of Senegal. The CHNEAR is the largest pediatric referral facility in the country and receives patients from all secondary health facilities nationwide. Senegal has an estimated population of 15.85 million and a gross national per capita income of US $950 [14]. The human immunodeficiency virus prevalence is low, 0.08 per 1000 persons, under 1% of the population The climate is tropical with well-defined dry and rainy seasons. The hot and dry Harmattan winds last from November until the onset of the rains in mid-May.

All children less than 5 years old who presented with suspected meningitis between 2010 and 2016 underwent a lumbar puncture as part of the routine diagnostic procedures. A suspected meningitis case was defined as sudden onset of fever (>38°C axillary or >38.5°C rectal temperature), with a combination of any of the following clinical symptoms: altered consciousness, stiff neck, sensitivity to light and bulging of the fontanelle if the child is less than 1 year old; a confirmed case was defined as any suspected meningitis case that is laboratory confirmed by culture or polymerase chain reaction (PCR) [15]. For all recruited cases, information on demographic, clinical, and vaccination status was captured by trained surveillance personnel.

A detailed methodology of all bacteriological methods of cerebrospinal fluid (CSF) collection has been reported elsewhere [16]. Briefly, CSF bacteriology was performed as previously described [17]. Following nucleic acid purification from CSF specimens, species-specific quantitative PCR (qPCR) for the detection of pneumococcus, meningococcus, and *H. influenzae* was performed [18–20]. A sequential multiplex qPCR assay for the detection of 21 different pneumococcal serotypes was performed as described by Pimenta et al [21]. Cerebrospinal fluid specimens with CT values less than 32 that could not be typed by the 21-multiplex qPCR were subjected to an in-house conventional PCR to detect additional serotypes (7C/7B/40, 15B/15C, 17F, 8, 9N/9L, 24A/24B/24F, 21, 35F/47F, 13, 10F/10C/33C, 34, 10A, 31) [16]. We employed the WHO pathogen-specific qPCR protocols to type/group *H. influenzae* and meningococcal [22].

Whole-genome sequencing was performed on genomic DNA extracted from 43 pneumococcal isolates using the Illumina HiSeq sequencer [23]. Sequencing reads from each isolate were mapped onto the *S. pneumoniae* ATCC_700669 serotype 23F reference genome using SMALT [24] and pseudo-genomes placed in a multiple-sequence alignment using custom scripts. Single nucleotide polymorphisms (SNPs) were called from the pseudo-alignment using SNP sites, and a maximum likelihood phylogeny was reconstructed with a general time reversible model using Randomized Axelerated Maximum Likelihood (RAxML) [9]. The phylogenetic tree was visualized and annotated using the interactive Tree of Life (iTOL) [25].

### Statistical Analysis

Baseline characteristics of the study participants were summarized using descriptive statistics. Proportions were based on the number of cases with available data for each variable. Univariate associations for clinical outcomes were assessed using χ ^2^ or Fisher’s exact tests where appropriate. Comparison of the clinical outcomes and pathogen and serotype distribution between the pre–PCV-13 era (2010–2013) and the post–PCV-13 era (2014–2016) were performed using χ ^2^ or Fisher’s exact tests. Data were analyzed using STATA version 13 (StataCorp).

Ethical approval was not a requirement in Senegal for routine meningitis surveillance including drug susceptibility testing of collected isolates as this was approved within the routine diagnostic algorithm at the Ministry of Health. However, informed consent was sought from the parents or guardians of the surveillance participants. Additionally, the surveillance received overarching ethical approval (SCC1188) by the joint MRC/The Gambia government ethics board that allowed the analysis of collected West African isolates at MRC Unit The Gambia.

## RESULTS

A total of 1013 suspected meningitis cases were recruited at the CHNEAR sentinel site between January 2010 and December 2016. The demographic and clinical characteristics of the children with suspected meningitis are summarized in Table 1. The median age of study participants was 9 months (interquartile range, 2.5–26 months), with most children aged less than 11 months (63% vs 37%, *P* < .001). Nearly one-fifth of the suspected cases had received antibiotic treatment prior to lumbar puncture. Outcome was reported for 90% (909 of 1013) of the children, and the case fatality ratio among them was 12.2%. More than half of the patients had a CSF specimen with a clear appearance (55%; 561 of 1010) as well as white blood cell counts of 10 cells/mm^3^ or less (58%; 568 of 1010) (Table 1).

**Table 1.** Patient Characteristics of Suspected Bacterial Meningitis Cases (N = 1013)

Characteristic and category	Total, n	Percentage
Age		
0–11 mo	639	63
12–23 mo	165	16
24–59 mo	208	21
Unknown	1	0
Sex		
Male	574	57
Female	439	43
Antibiotic before admission		
Yes	182	18
No	731	72
Unknown	100	10
Outcome		
Discharged alive	798	79
Died	111	11
Unknown	104	10
Cerebrospinal fluid appearance		
Clear	561	55
Turbid	214	21
Xanthochromic	114	11
Blood stained	124	12
White blood cell		
≤10 cells/mm^3^	585	58
>10 to 100 cells/mm^3^	176	17
>100 cells/mm^3^	234	23
Unknown	18	2

Overall, 99% (1010 of 1013) of CSF specimens were tested by at least 1 test (ie, rapid antigen tests, culture, or PCR). Almost one-tenth of the CSF specimens tested by PCR (115 of 1010) showed laboratory-confirmed bacterial meningitis. Pneumococcus was the major pathogen detected, accounting for 66% (76 of 115) of all confirmed cases, followed by meningococcus (26%; 30 of 115) and *H. influenzae* (9%; 9 of 115) (Figure 2).

**Figure 1.**
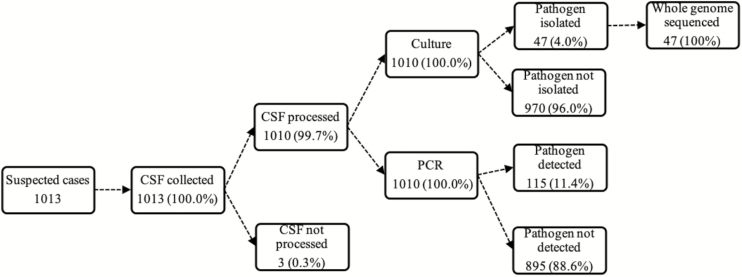
Summary of CSF collection and processing for invasive bacterial disease surveillance in Senegal (2010–2016). Abbreviations: CSF, cerebrospinal fluid; PCR, polymerase chain reaction.

Among the 39 confirmed pneumococcal meningitis cases, the dominant serotypes were 1, 5, 6A/6B, 7A/7F, 14, 19A, and 23F, all vaccine-type pneumococci (Figure 3). The highest proportions of pneumococci were detected in 2014, 1 year after introduction of PCV-13. However, a reduced number of pneumococcal meningitis cases were reported during 2015 (1 case) and 2016 (6 cases), the last 2 years of the surveillance. There was a moderate increase in the number of nonvaccine serotypes (Figure 3).

**Figure 2.**
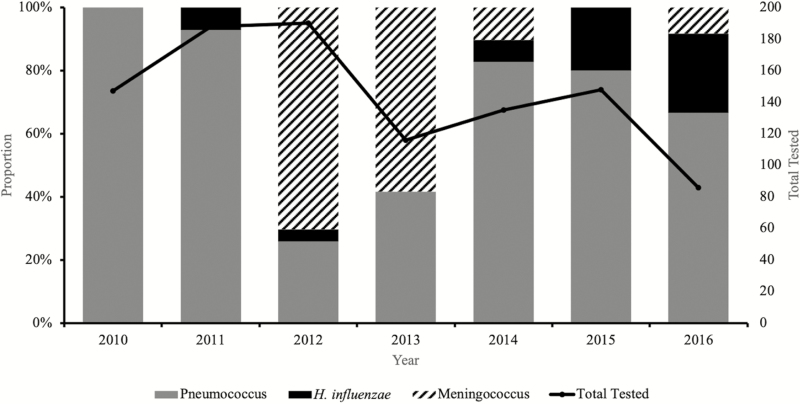
Distribution of pathogens associated with pediatric bacterial meningitis in Senegal (2010–2016).

**Figure 3.**
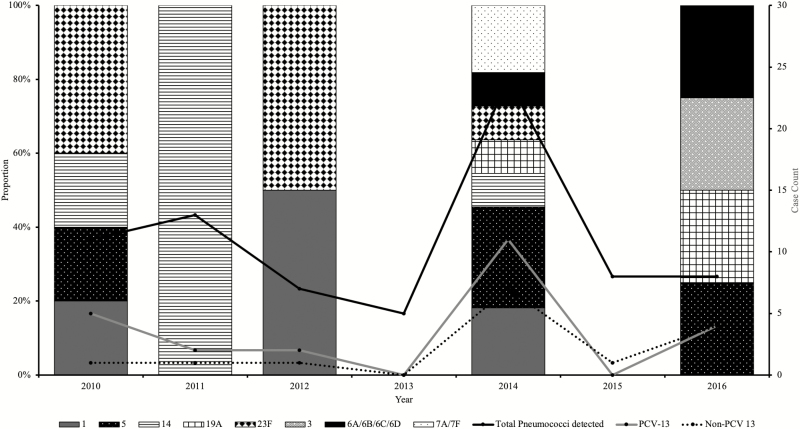
Temporal distribution of vaccine vs nonvaccine serotypes of pneumococcus by year (2010–2016). There was no serotyping result for all 5 pneumococcal meningitis cases in 2013; the sentinel site did not send CSF specimens for these cases to the WHO RRL. Abbreviations: CSF, cerebrospinal fluid; PCV-13, 13-valent pneumococcal conjugate vaccine; RRL, Regional Reference Laboratory; WHO, World Health Organization.

Of the 29 confirmed meningococcal meningitis cases, specimens for 12 were subjected to qPCR serogrouping and all belonged to serogroup W (NmW). *Haemophilus influenzae* type b accounted for nearly all (4 of 5) of all confirmed *H. influenzae* cases with a serotype result: 2 reported in 2014 and the other 2 in 2016. Only half (2 of 4) of the reported Hib cases received all 3 doses of Hib vaccine. The other *H. influenzae* isolate was type c (Hic).

Over half of all confirmed cases, 57% (66 of 115) were infants and pneumococci accounted for 76% (50 of 66). There were 7 pneumococcal meningitis cases among neonates (<28 days). In contrast, two-thirds of meningococcal and *H. influenzae* meningitis cases were among children 12 months of age and older. Case fatality rates were highest among infants aged 0–11 months (14%) compared with 11% and 7% among children aged 12 to 23 months and 24 to 59 months, respectively. Pneumococcus also accounted for 94% (17 of 18) of the deaths. The other death was a child with meningococcal meningitis. None of the children with *H. influenzae* meningitis died.

**Figure 4.**
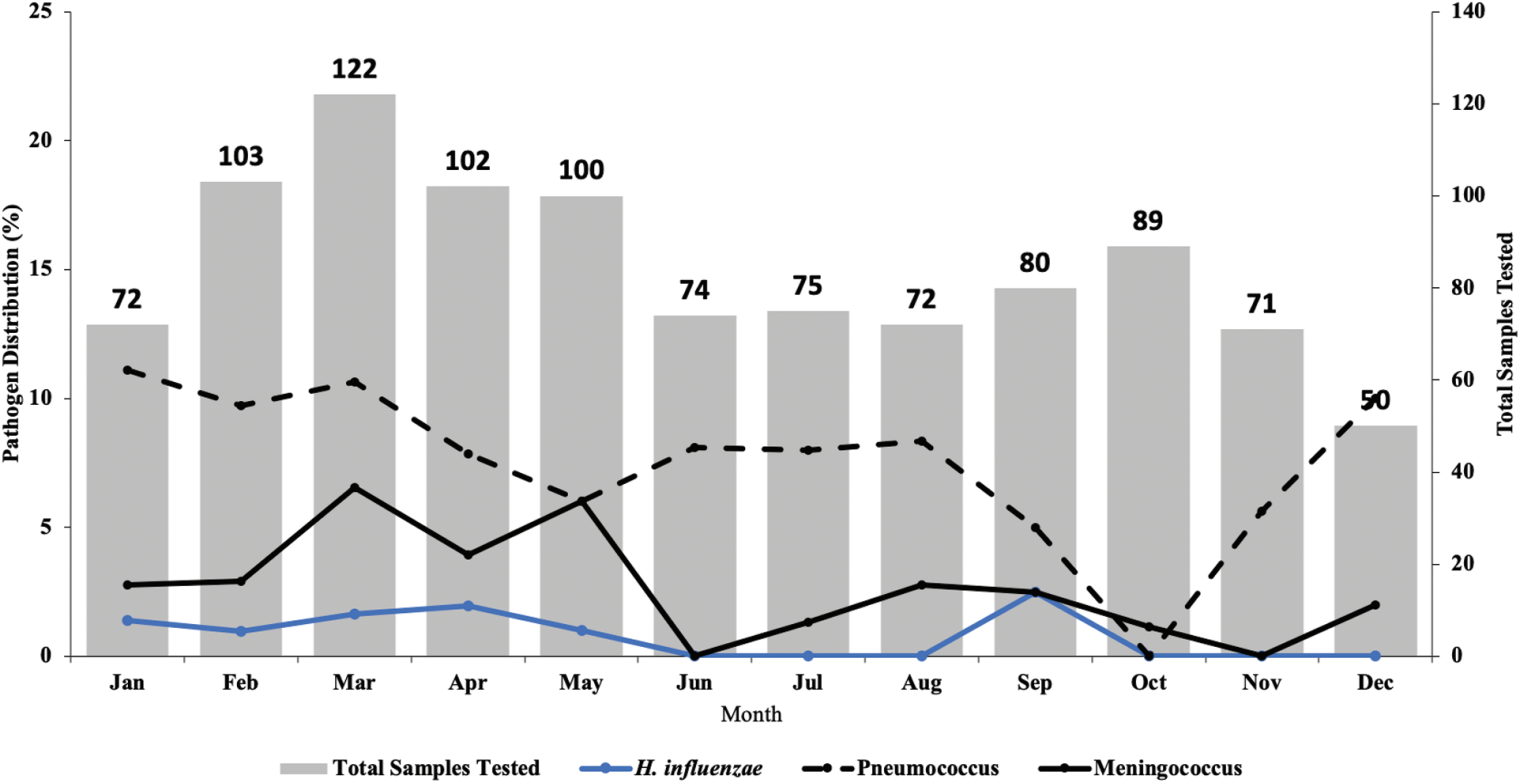
Seasonality of meningitis pathogens in the Senegal (2010–2016).

There was a decline in the number of suspected meningitis cases during the 7 years of surveillance, with just 86 suspected meningitis cases reported in 2016 compared with 150 in 2010 when the surveillance started (Figure 2). Meningitis pathogens were more frequently detected during the dry windy Harmattan season of January to May compared with the wet season (Figure 4).

**Figure 5.**
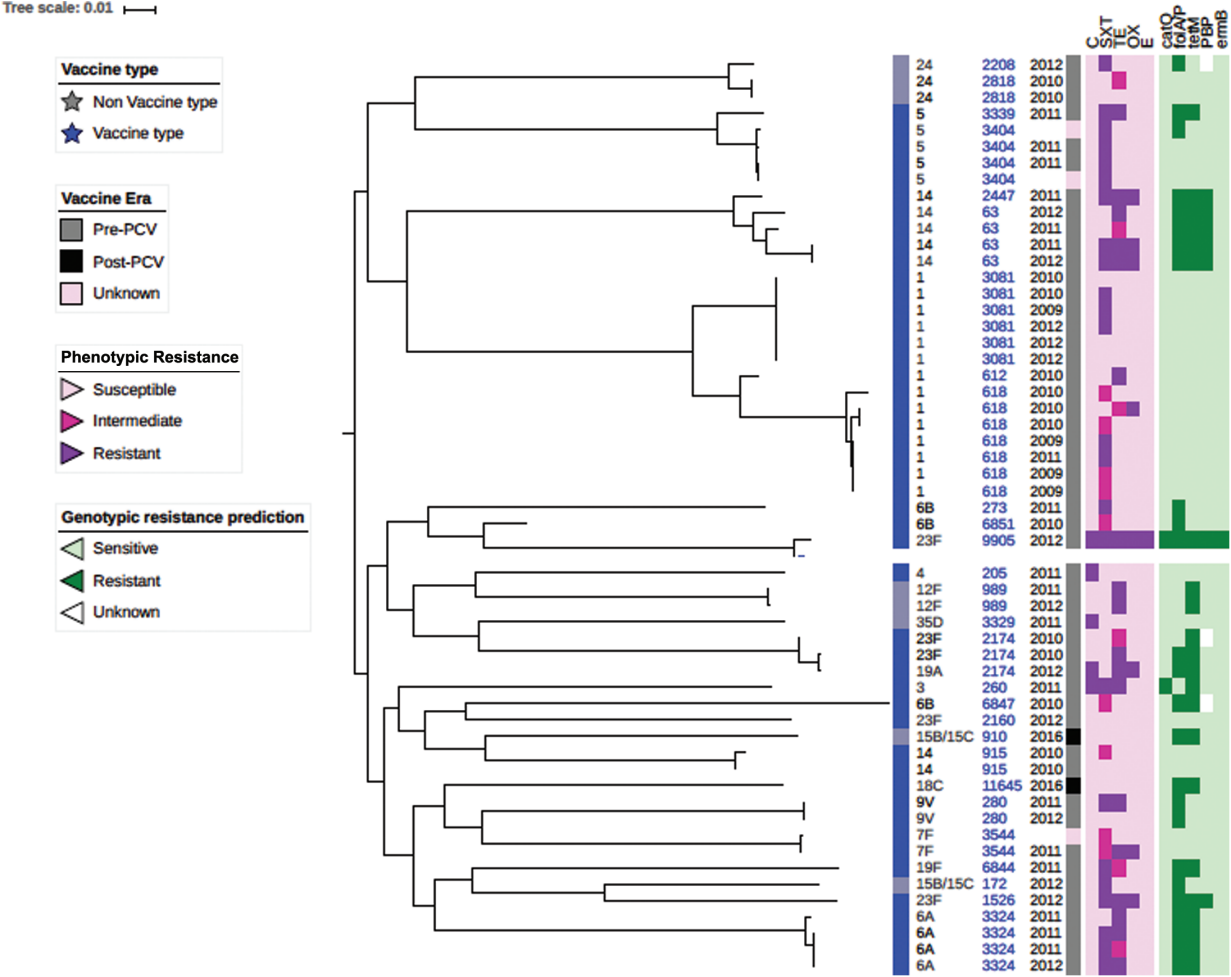
Phylogeny and antibiogram of pneumococcal isolates associated with childhood bacterial meningitis in Senegal. Pneumococcal isolates include those from 2009 (4 cases) to contextualize the isolates cultured from CSF specimens (39 cases). Resistance and intermediate resistance were confirmed by E-test. Abbreviations: C, chloramphenicol; CSF, cerebrospinal fluid; ERY, erythromycin; OX, oxacillin; PCV, pneumococcal conjugate vaccine; SXT, trimethoprim-sulfamethoxazole; TET, tetracycline.

The CHNEAR sentinel site sent a total of 55 pneumococcal isolates to the WHO RRL for confirmation, serotyping, antibiogram, and molecular characterization between 2010 and 2016 (Figure 5). These isolates were primarily from CSF of suspected cases of meningitis (47 cases) but also included 8 non-CSF isolates collected in 2009 for reference. One of the non-CSF isolates was cultured from a suspected meningitis case. Unfortunately, a case definition was not reported for the other 7 patients with a non-CSF pneumococcal isolate. Whole-genome sequencing analysis revealed 2 major clades (Figure 5)—clade 1: serotypes 1, 5, 14, and 24; and clade 2: all other serotypes including some of serotype 14. We found that isolates belonging to the same serotype clustered together, particularly the predominant invasive serotypes 1, 5, and 14. Sequence type (ST) 618 (n = 7) was the most common lineage among serotype 1 isolates, all of which were isolated prior to PCV-13 implementation in 2013. We noted an ST2174 lineage that included PCV-13 serotypes 19A and 23F, both of which were resistant to trimethoprim-sulfamethoxazole. Surprisingly, the other 23F serotype (ST9905) isolated was divergent to the 23F serotype (ST2174) that had clustered with serotype 19A, and this showed multidrug resistance to trimethoprim-sulfamethoxazole, chloramphenicol, erythromycin, and tetracycline. Among the isolates, high susceptibility to oxacillin and erythromycin was noted, while reduced susceptibility to trimethoprim-sulfamethoxazole and tetracycline, and to a lesser extent chloramphenicol, was observed. None of isolates was resistant to erythromycin. Compared with the pneumococcal serotype 1 ST3081 isolates, ST618 isolates were more resistant to trimethoprim-sulfamethoxazole: 50% (3 of 6 cases) vs 86% (6 of 7 cases), respectively.

## DISCUSSION

We present findings from 7 years of hospital-based sentinel surveillance of vaccine-preventable meningitis among children less than 5 years old in Senegal. Pneumococcus was the predominant etiologic agent among confirmed meningitis cases and was associated with high mortality in infants. There was a moderate decline in vaccine-type pneumococcal meningitis during the surveillance period. *Haemophilus influenzae* type b and NmW still remain important causes of bacterial meningitis among young children in Senegal despite the widespread use of conjugate vaccines.

In Senegal, Ba et al [26] reported (in descending order) serotypes 6B, 19F, 23F, and 14 as the prevalent serotypes in nasopharyngeal pneumococcal carriage and pneumococcal infection in children less than 5 years old between 2007 and 2008. This study confirmed a high rate of carriage and disease caused by pneumococcus serotypes contained within the current PCV formulations; the findings are also consistent with our surveillance data where we report serotypes 1, 5, 6A/6B, 7F/7A, 14, and 23F as the predominant serotypes in Senegal prior to PCV-13 introduction. The widespread use of PCVs has been shown to markedly reduce carriage of pneumococcal vaccine serotypes among both vaccinated and unvaccinated individuals [27–30]. PCV-13 was introduced into the Senegal Expanded Programme on Immunisation (EPI) in 2013. Although numbers were small, we showed a moderate reduction in the number of vaccine serotypes after PCV-13 introduction. This finding is consistent with findings from a recent population-based study of invasive pneumococcal disease in Senegal’s closest neighbor, The Gambia, which showed remarkable public health successes of PCVs associated with a 55% reduction in invasive pneumococcal diseases in children aged 2–59 months [31]. Compared with baseline, the researchers reported a 55% decrease in the incidence of invasive pneumococcal disease in the 2- to 23-month age group after PCV-13 introduction. This decrease was attributed to an 82% reduction in serotypes covered by the PCV-13 vaccine. A similar trend was observed in the 2- to 4-year age group, with a 56% reduction in PCV-13 serotypes. However, the incidence of non–PCV-13 serotypes in children aged 2–59 months increased by 47%, with a broad range of serotypes.

All serogrouped meningococci during the surveillance were NmW. Meningococcus serogroup W caused several sporadic meningitis cases in The Gambia in 1990s [32] and was also isolated during a serogroup A outbreak in Mali in 1994 [33]. Hossain et al [34] reported that NmW was the cause of the 2012 meningitis outbreak in The Gambia, where a total of 469 suspected cases were identified and 114 were confirmed NmW cases. Endemic NmW also persisted in South Africa between 2000 and 2005, which is outside the meningitis belt. Although the numbers we report are small, the predominance of NmW in Senegal supports the urgent need for a multivalent meningococcal vaccine in this region. Fortunately, the development of a thermostable pentavalent meningococcal conjugate vaccine (A, C, Y, W-135, X) by the Serum Institute of India is underway. It is expected that this formulation will be cheaper and rolled out in African countries in the next 5 years. We are hopeful that this vaccine will eliminate meningococcal disease caused by serogroups covered by the vaccine in the African meningitis belt.

Unsurprisingly, there was no NmA meningitis throughout the 7 years of surveillance. The MenAfriVac mass vaccination campaigns conducted in countries of the meningitis belt, including Senegal, are highly effective in prevention against serogroup A invasive meningococcal disease and carriage. However, there are concerns about how long this protection will persist as well as the emergence of disease caused by other serogroups not included in the formulation [35]. As with the MenAfriVac, vaccination campaigns targeting NmW could be conducted in high-risk groups or, better still, the Gavi and MVP could work toward supporting the development of multivalent vaccines including NmW in the routine EPI schedule for vulnerable populations in the meningitis belt.

*Haemophilus influenzae* type b was among the leading causes of bacterial meningitis in children younger than 5 years prior to the introduction of the Hib conjugate vaccine in Senegal [36]. The Hib vaccine introduced in 2005 in Senegal significantly reduced the incidence of Hib meningitis and pneumonia. Hospital-based surveillance data from the CHNEAR between 2002 and 2008 demonstrated the efficacy of Hib. The researchers reported a 98% reduction in the number of hospitalized Hib meningitis cases in 2008 compared with 2002. Similarly, introduction of Hib in The Gambia’s Western region showed that it was 95% effective in preventing meningitis and bloodstream infection and 100% effective in preventing pneumonia [37]. Another study by Oluwalana et al [38] between 2007 and 2010 found that the Hib conjugate vaccination as a primary 3-dose course in The Gambia remained highly effective in controlling invasive Hib disease. Recent data from Kenya showed that the use of a 3-dose primary series of Hib vaccine without a booster dose resulted in a significant and sustained reduction in Hib disease, suggesting that a booster dose is not currently required in Kenya. It is therefore concerning that Hib still causes bacterial meningitis in Senegal, with 4 cases detected throughout the surveillance period.

Whole-genome sequencing analysis of pneumococcal isolates showed that half of the serotype 1 strains belonged to ST618 [23], all of which were collected in the pre-PCV era. Post-PCV data from The Gambia has shown clonal replacement of serotype 1 ST618 by ST3081 [39]. Given the heterogeneity and recombinogenic nature of pneumococcus, continued surveillance is key to understanding the temporal evolution of pneumococcus and the potential for serotype replacement.

Our finding of seasonality in meningitis-causing pathogens agrees with the pattern seen in the meningitis belt, with peaks in the first 5 months of the year [8, 34, 40].

### Limitations

An important limitation of this surveillance is inherent in passive hospital-based identification of cases. It is likely that the data captured here are representative of urban/peri-urban children. These data may not be representative of rural populations at greater risk of disease. Children with the most severe disease may indeed never reach Dakar and would not be captured in this surveillance. In addition, it would have been very useful to know what percentage of the pneumococcal meningitis cases had received PCV. Unfortunately, these data were not available for such an analysis. This report would have been enriched by the inclusion of more detailed data on sequelae. For instance, up to one-quarter (238) of all suspected meningitis cases had missing data on sequelae because these data were not recorded routinely among suspected meningitis cases. Only a very small proportion (11.5%) of the suspected cases were confirmed as meningitis cases.

### Conclusions

Vaccine-preventable meningitis remains a major cause of child mortality and morbidity in Senegal. Enhanced surveillance for monitoring of vaccine impact and detection of meningitis is recommended for early detection of epidemics. There is a need to strengthen laboratory and surveillance capacity, which will enhance high-quality data capture on vaccine-preventable meningitis.

## Notes

***IBD*** ***Writing Group members.*** Brenda Kwambana-Adams, Madikay Senghore, Effua Usuf, Worwui Archibald, Uzochukwu Egere, Akram Zaman, Catherine Okoi, Florian Gehre, Leopold Tientcheu, Nuredin Ibrahim Mohammed, Felix Dube, Peter Ndow, Jason M. Mwenda, Sambou Suso, Sheikh Jarju, Dam Khan, Chinelo Ebruke, Rowan Bancroft, Jason Mwenda, and Martin Antonio.

***Author contributions.*** M. A. and J. M. M. established the World Health Organization (WHO)/AFRO-supported Pediatric Bacterial Meningitis Surveillance Network in West Africa. M. A. supervised the overall network, including setting up the sentinel surveillance system. M. A. S., A. D., A. T., P. M. F., B. D., I. D. B., B. D., O. D., O. N., and M. F. C. clinically investigated and recruited the patients at the sentinel sites, collected demographic data, performed microbiological testing at sentinel sites, and shipped cerebrospinal fluid and bacterial isolates to WHOCC at the Medical Research Council (MRC) Unit The Gambia for confirmatory testing and molecular analysis at MRC Unit The Gambia supervised by B. A. K.-A. and M. A. B. A. K.-A. and M. A. developed the analysis plan, contributed to analysis and interpretation of data along with the IBD writing group. F. S. D., B. A. K.-A., and M. A. drafted the paper along with M. A. S. All authors contributed to the interpretation of the findings and the writing of the final manuscript.

***Acknowledgments.*** The authors thank the WHO Country Office of Senegal and WHO IST for coordination, advice, and support throughout the project. We thank the surveillance participants and their families in Senegal and the staff members and students at the WHO Regional Reference Laboratory, MRC Unit The Gambia at the London School of Hygiene and Tropical Medicine, as well as the members of the IBD writing group for their advice and support.

***Financial support.*** Financial support for sentinel site surveillance was provided by the Senegal Ministry of Health, Gavi - the Vaccine Alliance, through a grant to the WHO for the African Paediatric Bacterial Meningitis Surveillance Network.

***Supplement sponsorship.*** This supplement was supported with funds from Gavi, the Vaccine Alliance through The World Health Organization and the CDC Foundation, and The Medical Research Council Unit The Gambia at the London School of Hygiene and Tropical Medicine.

***Potential conflicts of interest.*** The authors: No reported conflicts of interest. All authors have submitted the ICMJE Form for Disclosure of Potential Conflicts of Interest. Conflicts that the editors consider relevant to the content of the manuscript have been disclosed.
